# Lack of Association between the* IL6R* Gene Asp358Ala Variant (rs2228145), IL-6 Plasma Levels, and Treatment Resistance in Chilean Schizophrenic Patients Treated with Clozapine

**DOI:** 10.1155/2019/5601249

**Published:** 2019-06-25

**Authors:** Alvaro Cavieres, Carolina Campos-Estrada, Yanneth Moya, Rocío Maldonado, René González-Vargas, María Leonor Bustamante, Pablo R. Moya

**Affiliations:** ^1^Departamento de Psiquiatría, Escuela de Medicina, Universidad de Valparaíso, Chile; ^2^Escuela de Química y Farmacia, Facultad de Farmacia, Universidad de Valparaíso, Chile; ^3^Centro de Investigación Farmacopea Chilena, Universidad de Valparaíso, Chile; ^4^Instituto de Fisiología, Facultad de Ciencias, Universidad de Valparaíso, Chile; ^5^Departamento de Psiquiatría y Salud Mental Norte, Programa de Genética Humana, Instituto de Ciencias Biomédicas, Facultad de Medicina, Universidad de Chile, Chile; ^6^Centro Interdisciplinario de Neurociencia de Valparaíso CINV, Universidad de Valparaíso, Chile

## Abstract

Alterations in neuroinflammatory processes have been suggested to contribute to the development of Schizophrenia (SZ); one component of the inflammatory system that has been linked to this disorder is interleukin-6 (IL-6). The minor allele of rs2228145, a functional polymorphism in the IL-6 receptor gene, has been associated to elevated IL-6 plasma levels and increased inflammatory activity, making it an interesting candidate to study as a possible factor underlying clinical heterogeneity in SZ. We studied a sample of 100 patients undergoing treatment with clozapine. Their symptoms were quantified by Brief Psychotic Rating Scale; those with the lowest scores (“remitted”) were compared with the highest (“clozapine treatment resistant”). We determined allelic frequencies for rs2228145 and IL-6 plasma levels. Our results do not support a role of IL-6 in response to treatment with clozapine. Further studies accounting for potential confounding factors are necessary.

## 1. Introduction

Schizophrenia (SZ) may be etiologically related to a chronic neuroinflammatory process, with greater inflammatory activity in affected individuals with active psychosis as well as in treatment-resistant cases [1–3]. In this regard, most evidence comes from the observed higher risk of presenting SZ in subjects whose mothers suffered infections during pregnancy, with activation of the maternal immune response [4], and from findings of elevated levels of immune system components in SZ-affected individuals [5]. Variations in complement activity, in turn, have been linked to changes in synaptic pruning in mice, providing an explanatory mechanism for the role of inflammation in SZ [6].

Several inflammatory mediators have been proposed to be implicated in SZ, including interleukin 6 (IL-6) [7, 8]. IL-6 exerts its proinflammatory effects via trans-signaling, binding to the soluble form of the IL-6 receptor (sIL-6R), which is generated by mRNA alternative splicing and partial proteolysis [9]. The coding single nucleotide polymorphism (SNP) rs2228145 of IL-6R gene leads to a substitution of aspartic acid to alanine in position 358 (Asp358Ala variant). The minor allele of rs2228145 (Ala) has been associated with higher sIL-6R levels and circulating/plasma IL-6 levels [9–11].

Studies on the potential role of rs2228145 in SZ, however, are inconclusive. One genetic study described a significant association between the Ala allele and the disorder, with the authors suggesting that it could partly explain the increased concentration of the cytokine reported in patients [12]. However, increased IL-6 levels without significant change in sIL-6R levels have been reported in SZ individuals [13, 14].

To date, rs2228145 has not emerged in any genome wide association study (GWAS) of SZ. Nevertheless, rs2228145 has been associated with significance beyond GWAS threshold to 8 phenotypes related with inflammation, including allergy, dermatitis, and rheumatoid arthritis [15–17]. Furthermore, it has been recently shown that rs2228145 influences the blood and cerebrospinal fluid levels of sIL-6R [18].

Thus, considering the functional role of the rs2228145 polymorphism on a key factor in inflammatory response, along with the evidence of inflammatory process as one of the mechanisms underlying SZ, we sought to contribute to clarify its possible association with specific symptoms or conditions, like treatment-resistance.

At least one third of SZ patients are resistant to pharmacological therapy with antipsychotics. In case of inadequate response to first-line antipsychotics, the most accepted strategy is treatment with clozapine. Approximately, one third of the patients treated with clozapine will present persistent symptoms and thus will be considered resistant. The lack of response to clozapine could be related to an ongoing low-grade inflammation [19], which, in turn, may be mediated by genetic variants including rs2228145. Thus, we aimed to examine the association between rs2228145 and circulating IL-6 plasma levels with resistance to clozapine in a sample of SZ patients treated at the outpatient section of the Psychiatric Hospital del Salvador in Valparaíso, Chile.

## 2. Materials and Methods

This study was reviewed and approved by the Ethics Committees of the Valparaíso-San Antonio Health Service and the Clinical Hospital of Universidad de Chile. Written informed consent was provided by all participants. This study has an observational, analytical, case-controlled design.

### 2.1. Subjects

Patients were recruited among consultants to the outpatient clinic of the Psychiatric Hospital del Salvador, Valparaíso. Patients who have a diagnosis of SZ (according to DSM-IV) and have failed to improve after two courses of antipsychotics at adequate doses for 6 to 8 weeks, with at least one of them of second generation, are admitted into the clozapine program. To be included in the present study, the patients must have been receiving clozapine at a minimum dose of 300 mg per day, for at least six months. Because of the availability of the consultants to the clinic, we did not perform a sample size calculation, but rather included as many of the patients as possible. One hundred individuals (representing 75% of the total population of consultants to the clinic) agreed to participate and provided a DNA sample.

Response status was established according to the classification, as remitted or resistant, using the Spanish version of the Brief Psychotic Rating Scale (BPRS). Subjects were considered to be in remission if they had scores of mild or less in all the following BPRS items: 4,7,8,11,12,15,16. On the other hand, resistance to clozapine treatment was defined as an overall score ≥ 45 on the BPRS scale and/or ≥ 4 (moderate) on two or more items of psychotic symptoms, after at least six months of treatment, with doses ≥ 300 mg of clozapine. Subjects whose score was intermediate between these two extremes were not considered in the analysis.

A control group of 48 subjects was also recruited to serve as a general comparison at the Clinical Hospital of Universidad de Chile. Mental disorders were ruled out using the Spanish version of the MINI International Neuropsychiatric Interview (MINI) interview [13]. A posteriori calculation based on the guidelines established by Hong and Park [20] shows that our sample has an 80% power to detect differences with an Odds Ratio of 2.

### 2.2. Methods

Genomic DNA was extracted using a commercial Blood DNA kit (Qiagen, USA). Concentration and purity were determined by spectrophotometry; DNA integrity was confirmed with beta-actin PCR amplification. DNA samples were stored at -80°C until their use. Allelic determination of rs2228145 was carried out using real-time TaqMan SNP genotyping (ID assay: C__16170664_10; Thermo Scientific, MA, USA) using 10 ng of genomic DNA. IL-6 plasma levels of IL-6 were determined from peripheral venous blood samples (forearm vein) by standard venipuncture. Blood samples were collected between 7:00 and 9.00 h into vacuum blood-collecting tubes containing EDTA 1 mg/mL; plasma samples were obtained by centrifugation immediately for 10 minutes at 3500 rpm. The plasma was then stored frozen until further analysis. Quantitative determination of IL-6 was performed through quantitative enzyme-linked immunosorbent assay using a commercially available kit (R&D Systems USA, Minneapolis) according to manufacturer's instructions. 100 *μ*L of standards or plasma sample was added to each well and incubated for 2 hr at room temperature. A calibration curve was made using Human IL-6 Standard (in Assay buffer 1X) at 300, 100, 50, 25, 12.5, 6.25, 3.13, and 0 pg/mL. After washing, 200 *μ*L of human IL-6 conjugate (enzyme-linked polyclonal antibody specific for human IL-6) was added to the wells followed by a 2 hr incubation at room temperature. Then, 200 *μ*L of substrate solution was added to each well and incubated for 20 minutes at RT. The reaction was stopped with 50 *μ*L of stop solution and the colour intensity was measured. Absorbance was interpolated from the calibration curve. The sensitivity was 0.7 pg/mL in a range of 0-300 pg/mL with intra-assay and interassay coefficients of 4,2% to 7%, respectively. There were no undetectable values. All samples were over the detection limit.

### 2.3. Statistical Analysis

Quantitative results are described with measures of central tendency and dispersion; frequency and proportions are used for qualitative data. Statistically significant differences in the allelic and genomic frequency between groups were determined with Chi^2^ Test or Fisher's Exact Test. Hardy-Weinberg equilibrium was assessed using the “Genhw” function of the STATA software, considering p <0.05 as not consistent with equilibrium.

A histogram showed that the clinical data of this study do not follow a normal distribution; therefore Mann-Whitney U test was used to determine differences in IL-6 values between groups; correlations between variables were analysed with Spearman test. Association between genotypes and IL-6 value was determined with the Kruskal Wallis test.

All analyses were performed with Stata 15 SE software; statistical significance was set at 0.05.

## 3. Results

Blood samples were obtained from 100 SZ patients (mean age= 42.67, SD 11.46, 65% male gender) and 48 control subjects (mean age= 26.16 years, SD 15.32, 48% male gender).

All genomic DNA samples were successfully genotyped. Allelic and genotyping frequencies of both SZ patients and controls were consistent with Hardy-Weinberg equilibrium. We found no differences in genotypic or allelic frequencies of rs2228145 between the SZ patients and controls (see Table 1). Next, we compared patients with BPRS scores on the extreme high (“resistant to clozapine,” n=41) and extreme low (“remitted,” n=24) ranges. Patients with intermediate scores in BPRS (n=35) were excluded from the following analysis. Table 1 depicts genotypic and allelic frequencies for these two groups, as well as their median IL-6 levels, and the genotypic and allelic frequencies of controls. No significant differences were found in the frequencies of resistant vs. remitted patients, or in the frequencies of resistant patients vs. controls.

A correlation analysis between IL-6 plasma levels and scores in the positive and negative subscales and total scores in the BPRS of the total sample of patients did not yield statistically significant results either (data not shown). Further, as shown in Table 2, IL-6 plasma levels were found similar for all rs2228145 genotypes.

## 4. Discussion

Our results do not confirm the initial hypothesis of a higher frequency of the rs2228145 Ala allele in our sample of SZ subjects, or increased IL-6 levels in the clozapine treatment-resistant subgroup subjects, although there was a trend to a higher frequency of the A allele in clozapine treatment resistant individuals. Due to safety concerns, clozapine is not considered a first-line treatment, and according to most guidelines for SZ, all patients must undergo two trials of different antipsychotics before starting clozapine. Thus, it is possible that our sample represents a subpopulation among SZ patients that is more severe (and more homogeneous) than the general population of patients.

Our sample has adequate statistical power to detect differences in variants with a moderate effect on the phenotype. However, our sample size is limited for detecting small effects, and this could be an explanation of the lack of association reported here. Nevertheless, since we selected the extreme cases of response to clozapine for comparison, we expect that genetic variants influencing this trait would have an effect large enough to be detected by our design. Furthermore, because antipsychotic resistance is a significant clinical problem and our country has a network of health care specifically for these patients, our study highlights the need to integrate data from the national networks of clozapine and atypical antipsychotics pharmacovigilance protocols in our country.

Future studies should also consider the impact of additional relevant* IL6R *gene polymorphisms to further investigate possible haplotype associations, as well as to search for genetic variation and circulating levels of IL-6R. To the best of our knowledge, this is the first study in Chilean population addressing genetic factors affecting IL-6 levels and treatment resistance in SZ individuals. Ancestry genetic factors that could be specific to our population should also be included in future studies, to allow proper comparison with studies performed worldwide.

Likewise, important factors affecting plasma IL-6 levels that were not controlled in this study should be considered, especially those of metabolic origin, frequently altered in people with SZ [21]. Also, it is known that clozapine administration may indeed affect circulating interleukin levels, which could nullify an initial elevation of interleukin [22]. Similarly, it has also been reported that treatment with other neuroleptics may decrease the levels of sIL-6R [23].

In conclusion, our study does not provide support to a role for the IL-6R rs2228145 (Asp358Ala) variant of the* IL6R* gene in the responsiveness to clozapine in SZ affected individuals. Efforts should be made to increase our sample size and control additional factors affecting interleukin levels, as discussed above.

## Figures and Tables

**Table 1:** IL-6 plasma levels and rs2228145 genotypic and allelic frequencies for SZ patients and controls. p: *p* value for the comparison of each group resistance vs. remission and resistance vs. control.

	Median IL-6 plasma levels (pg/ml) [Interquartile range]	Genotypic frequencies n (%)				Allelic frequencies n (%)		
		CC	AC	AA	p	C	A	p
Resistance	1.91 [1.35-3.1]	8 (34.8)	11 (43.5)	5 (21.7)		19 (47.5)	21 (52.5)	

Remission	2.25[1.20-3.22]	14 (36.1)	23 (55.6)	4 (8.3)	0.35	51 (62)	31 (38)	0.17

Control	N.A.	20 (42)	21 (44)	7 (15)	0.71	61 (63.5)	35 (36.4)	0.12

**Table 2:** IL-6 plasma levels according to rs2228145 genotype.

		Genotype		
	AA	AC	CC	p
Median IL-6 plasma levels (pg/ml) [Interquartile range]	1.833 [1.132-3.28]	2.395 [1.605-3.197]	1.497 [1.017-3.28]	0.46*∗*

## Data Availability

IL-6 plasma levels, rs2228145 genotype, and BPRS status data can be requested from the corresponding authors.
